# Development and Characterization of Heparin–Pullulan Liposomal Nano-Gel for Enhanced Silymarin Delivery in Dementia Therapy: In Vivo Evaluation in Albino Mice

**DOI:** 10.3390/pharmaceutics18030348

**Published:** 2026-03-11

**Authors:** Aamir Mushtaq, Hamid Saeed Shah, Sairah Hafeez Kamran, Umar Farooq Gohar, Carmen Daniefla Neculoiu, Petru Cezario Podasca, Marius Alexandru Moga, Andrada Camelia Nicolau

**Affiliations:** 1 Department of Pharmaceutical Sciences, Government College University Lahore, Lahore 54000, Pakistan; 2 Institute of Pharmaceutical Sciences, University of Veterinary and Animal Sciences Lahore, Lahore 54000, Pakistan; hamid.saeed@uvas.edu.pk; 3 Institute of Pharmacy, Faculty of Pharmaceutical and Allied Health Sciences, Lahore College for Women University, Lahore 54000, Pakistan; sairah.hafeez@lcwu.edu.pk; 4 Institute of Industrial Biotechnology, Government College University Lahore, Lahore 54000, Pakistan; dr.mufgohar@gcu.edu.pk; 5 Faculty of Medicine, Transilvania University of Brasov, 500036 Brasov, Romania; caesarpodaska@yahoo.com (P.C.P.); moga.og@gmail.com (M.A.M.); andrada@unitbv.ro (A.C.N.)

**Keywords:** nano-carrier system, controlled drug release, neuroprotection, oxidative stress, cognition

## Abstract

**Background/Objectives**: Dementia remains one of the major global health challenges of the modern era. Researchers worldwide continue to seek effective therapeutic strategies to combat this neurodegenerative condition. Silymarin is a natural compound with strong neuroprotective and antioxidant properties that holds great potential for dementia management; however, its poor aqueous solubility and limited ability to cross the blood–brain barrier (BBB) have restricted its clinical application. This study focused on the formulation and evaluation of a heparin–pullulan silymarin liposomal (HPSL) nano-gel to enhance the neuroprotective efficacy of silymarin, with potential for improved brain targeting effects. **Methods**: The HPSL nano-gel was synthesized using the thin-film hydration technique and optimized based on entrapment efficiency, particle size distribution, zeta potential, and in vitro release kinetics. The neuroprotective efficacy of the HPSL nano-gel was evaluated in mice using behavioral evaluations, biochemical quantification of oxidative stress markers, evaluation of cholinergic enzyme activity and detailed histopathological examination of brain tissues. **Results**: Morphological characterization using scanning electron microscopy (SEM) confirmed a uniform nano-scale structure. The optimized formulation (HPSL-3) exhibited a particle size of 406.07 ± 19.33 nm, zeta potential of −23.72 ± 7.64 mV and an entrapment efficiency of 73.53 ± 12.05%, indicating good colloidal stability and efficient drug loading. The in vitro release profile followed non-Fickian diffusion kinetics, suggesting sustained drug release behavior. Behavioral studies in scopolamine-induced amnesic mice (elevated plus maze, hole board, and light/dark paradigms) demonstrated significant (*p* ≤ 0.001) improvements in learning and memory retention. Biochemical analyses showed increased levels of ChAT, SOD, CAT, and GSH, along with decreased AChE and MDA levels, supporting the neuroprotective potential of the formulation. Histopathological evaluation revealed marked attenuation of neuronal degeneration, inflammation, and edema (HAI = 4) compared to the scopolamine-treated group (HAI = 11). **Conclusions**: Overall, the HPSL-2 formulation effectively enhanced silymarin delivery across the BBB, demonstrating potent antioxidant, neuroprotective, and cholinergic modulatory effects. These findings suggest that HPSL-2 represents a promising nano-carrier system for the management of dementia and other oxidative-stress-related neurological disorders.

## 1. Introduction

Dementia is a progressively occurring neurological disorder characterized by cognitive impairment and loss of memory and other intellectual functions of the human brain. It poses an increasing threat to global health in the modern era [1]. There are multiple causes of dementia, with Alzheimer’s dementia being the most common. Vascular dementia, frontotemporal dementia, Lewy body dementia and Alzheimer’s dementia are among the most well-known types of dementia [2]. The pathophysiology of dementia is very complex depending upon the particular cause [3]. The cholinergic hypothesis best describes the pathogenesis of dementia, stating that the loss of cholinergic neurons due to neurodegeneration lowers acetylcholine below the normal level in the human brain [4]. Aging and oxidative stress are two leading causes of neurodegeneration in the brain [5]. Dementia has become a significant global health issue, with a prevalence of almost 55 million people worldwide. This situation is becoming more alarming day by day, and the WHO has estimated that by 2050 this figure will surpass 150 million [6,7]. Dementia not only has a very negative impact on the quality of life of individuals suffering from the condition but also affects the routine functioning of their families and caregivers. The progressive cognitive decline of patients ultimately leads to physical, emotional, behavioral and financial disturbances within their families [8]. One of the major hindrances to the management and care of dementia patients is the lack of a timely, accurate diagnosis. Healthcare professionals face difficulties in ruling out dementia, since no definitive diagnostic tools are available for comprehensive cognitive assessment [9]. The second major problem is the unavailability of disease-modifying therapies that can slow the progression of dementia. The medicines currently used for the management of dementia can only provide temporary symptomatic relief while modestly improving the quality of life of the patients [10]. Currently, anticholinergic drugs (galantamine, donepezil, rivastigmine and tacrine), NMDA receptor blockers (memantine), and some antidepressants and antipsychotics are prescribed to improve memory and other cognitive functionalities, along with psychological and behavioral symptoms [11,12]. The major limitation of these medications is that patients often experience serious side effects, leading to poor treatment compliance and further worsening of symptoms. Therefore, scientists are continuously searching for therapeutic regimens that provide better efficacy with minimum risks of adverse effects. Consequently, growing attention has been directed toward natural products, which are generally considered safer and less toxic when used at therapeutic doses. Silymarin is a well-known herbal compound originally extracted from *Silybum marianum* that is commonly known as the milk thistle plant [13]. Silymarin is most commonly known for its hepatoprotective activity in management of fatty liver disease, cirrhosis and hepatitis [14]. It exhibits excellent antioxidant activity and is therefore widely used in the management of chronic inflammation (skin ulcers, inflammatory bowel disease and arthritis), neurodegenerative disorders, cancers (lung, liver, colon, prostate, breast, etc.), diabetes and skin health (rosacea, acne and healing wounds) [15]. Silymarin is a very potent antioxidant and its role in the amelioration of dementia has been well-explored in scopolamine-induced hyperamnesic rat models [16]. The major hindrance in the therapeutic use of silymarin for neurological disorders is its poor ability to exert neuroprotective effects in the brain, partly due to limited permeability across the blood–brain barrier [17]. Similarly, the pharmacotherapy of neurological disorders including dementia faces a major challenge of blood–brain barrier hindrance, which restricts the transportation of many therapeutic agents to brain parenchymal cells. Consequently, the use of conventional drug delivery systems results in sub-therapeutic drug concentrations at the target site. To achieve the desired therapeutic effect, higher doses are often required, which may lead to systemic side effects. To overcome this limitation, nano-carrier-based drug delivery systems, such as liposomes, can be used to enhance not only biocompatibility but also the neuroprotective efficacy of therapeutic agents [18,19]. However, hydrophilic polymers like pullulan can also be incorporated to facilitate the receptor-mediated transcytosis across the blood–brain barrier. The liposomal core can be combined with a polysaccharide-based shell to construct a versatile nano-gel platform. This approach improves drug stability and release characteristics while enabling the delivery of therapeutic concentrations to the brain for the effective management of dementia [20].

The current study is designed to develop and characterize a heparin–pullulan liposomal nano-gel encapsulating silymarin for effective drug delivery to the brain. Detailed behavioral, biochemical and histopathological studies in mice are performed to evaluate the brain-targeting efficiency and therapeutic potential in a dementia model. This research provides valuable insights into nano-gel-based strategies for overcoming blood–brain barrier limitations and offers a promising platform for the development of effective therapies for dementia.

## 2. Materials and Methods

### 2.1. Chemicals

Silymarin (purity ≥ 98%), heparin (low molecular weight, ~6 kDa, pharmaceutical grade), pullulan (molecular weight ~200 kDa), phospholipids (soy lecithin, DSPC), cholesterol, dialysis membranes (MWCO 10 kDa), cell culture media (DMEM), fetal bovine serum (FBS) and other cell culture reagents were procured from Sigma-Aldrich, Lahore, Pakistan). Ethanol (analytical grade) and chloroform were procured from Merk Lahore Pakistan. All other chemicals and solvents used in this study were of analytical grade and utilized without further purification.

### 2.2. Preparation of HPSL Nano-Gel

The HPSL nano-gel was prepared using a modified thin-film hydration method followed by integration into a heparin–pullulan hydrogel network. Initially, phospholipids (DSPC, 100 mg), cholesterol (25 mg), and silymarin (10 mg) were dissolved in a 10 mL mixture of chloroform and ethanol (2:1, *v*/*v*) [21,22].

The organic solvent was evaporated under reduced pressure using rotary evaporation at 40 °C and 100 rpm to form a thin, uniform lipid film on the inner wall of a round-bottom flask. To ensure complete removal of residual solvents, the flask was kept under vacuum for 12 h. The dried lipid film was then hydrated with 10 mL of phosphate-buffered saline (PBS, pH 7.4) preheated to 37 °C, under continuous stirring at 200 rpm for 30 min. The resulting multilamellar vesicles (MLVs) were subsequently sonicated using a probe sonicator (Stalwart, Van Nuys, LA, USA) set at 40% amplitude, applying 10 cycles of 30 s ON and 30 s OFF, for a total of 5 min, leading to the formation of nano-sized unilamellar liposomes with an approximate size of 150 nm.

For nano-gel formation, pullulan (50 mg) and heparin (30 mg) were dissolved in 5 mL of PBS (pH 7.4) at 25 °C under continuous stirring at 500 rpm for 1 h, allowing the formation of a uniform polymeric solution. The prepared liposomal dispersion was then gradually introduced into the polymer matrix under constant stirring at 300 rpm for 3 h to facilitate electrostatic interactions between the negatively charged heparin and the positively charged liposomes. This process resulted in the stable integration of liposomes within the nano-gel network. Finally, HPSLs were centrifuged for 20 min at 8000× *g* by using a centrifuge machine (Hettich EBA 200S, Singapore, Singapore) and extracted HPSL nano-gel was lyophilized in a single-chamber LSCplus Martin Christ^T.M^ freezer (Osterode am Harz, Germany) by keeping them at −40 °C overnight. The final HPSL nano-gel formulation was stored at 4 °C for further characterization.

### 2.3. Entrapment Efficiency

The procedure that was previously established underwent only minor modifications for the calculation of the entrapment efficiency (EE) percentage [23]. A total of 500 mg of HPSL nano-gel, which was equivalent to 5 mg silymarin, was solubilized in 5 mL of PBS having pH of 7.4. After that, the mixture was introduced into a dialysis membrane and was agitated for 1 h at 37 °C. A magnetic stirrer was used for this purpose at a speed of 100 rpm. After 0 and 1 h, a 5 mL sample was drawn from the mixture and the absorbance was read on UV–visible spectrophotometer (Shimadzu UV 1900i, Tokyo, Japan) at a wavelength of 239 nm.

The % EE was calculated by using the following equation:

$$EE\;\left(\%\right)=\frac{Total\;silymarin\;added-silymarin\;in\;supernatant}{Total\;silymarin\;added}\times 100$$

### 2.4. Assessing Hydrodynamic Dimension and Zeta Potential: A Measurement Investigation

Ultrapure distilled water was used to find the hydrodynamic size of HPSL nano-gel. The particle sizes and zeta potential were examined by using a Malvern Zetasizer Nano ZS (Cambridge, UK) [24].

### 2.5. Visual Examination Using Scanning Electron Microscopy (SEM)

Scanning electron microscopy (SEM) was performed by a Hitachi S-4700 scanning electron microscope (Hitachi High Technologies Coorporation, Tokyo, Japan). It was activated at an acceleration voltage between 10 and 20 kV and the sample was solubilized in ethanol and was loaded as a fast dispersion. It was then deposited onto freshly washed silicon wafers for the purpose of desiccation. To enhance the conductivity of the material, a gold-sputter covering was used on the HPSL nano-gel samples [25].

### 2.6. Release Kinetics of Silymarin

The liberation of silymarin from HPSL nano-gel was observed by using a well-known methodology with the aim of developing kinetic models [26]. In short, 500 mg silymarin–NS was dispersed in 5 mL of pH 7.4 phosphate-buffered saline supplemented with hyaluronidase (50 U/mL). The mixture was placed on a dialysis membrane and immersed in 100 mL of phosphate-buffered saline (pH 7.4) with constant agitation by adjusting the agitator to 75 rpm and temperature to 37 °C. The release of silymarin was determined periodically by measuring the absorbance at 239 nm by using a UV-Vis spectrophotometer (Shimadzu UV 1900i, Shimadzu Corporation, Tokyo, Japan). The release process of pure silymarin from NS was determined by using DDSolver software v1.0 (2010) and release-kinetics-based mathematical models.

### 2.7. Animals

Male Swiss albino mice having weight 27 ± 3 g were used for assessment of behavioral and biochemical parameters. Freshly bred, healthy animals were selected in accordance with the criteria established by the Institutional Animal Ethics Committee and allocated to different experimental groups based on the study design. They were kept in polycarbonate cages under standard environmental conditions (temperature 25 ± 2 °C; humidity 50–55% and light/dark ratio of 12 h each). The animals were first acclimatized to the lab environment for one week and supplied with standard palette diet and water ad libitum throughout the study. All the animals were initially trained for one week before the start of behavioral studies. Formal approval regarding ethics of animal experiments was obtained, vide voucher no AEC/Pharm-GCU/0071-1A.

#### Study Design and Dosing of Animals

The animals were randomly divided into seven groups (G-I to G-VII), with six animals in each group (n = 6). The study design, as outlined in Table 1, describes the treatment protocols followed for behavioral and biochemical evaluations. Prior to the main study, a pilot experiment was conducted in albino mice to determine appropriate dose levels. Dose selection (20 and 40 mg/kg) was based on an acute tolerability study of HPSL-2 without silymarin in male albino mice (n = 6 per group), administered at doses ranging from 20 to 60 mg/kg. The animals were closely observed for behavioral changes, clinical signs of toxicity, and mortality. The 40 mg/kg dose was well tolerated, with no observable adverse effects up to two weeks post-administration and was therefore selected as the highest dose for the experimental study. The study design mentioned in Table 1 indicates the details of how the animals were treated for behavioral and biochemical studies.

### 2.8. Behavioral Studies

Three well-known paradigms, elevated plus maze, light/dark test and hole board phenomenon, were selected for the assessment of behavioral activity. Animals were given treatment according to the study design (Table 1) for one week and then all the animals were individually subjected to the above paradigms on day 7 and day 8 of treatment. Initial transfer latencies, retention transfer latencies and inflexion ratios were calculated through experimentation using an elevated plus maze (EPM). The EPM apparatus consisted of two open arms, two closed arms, and a central platform elevated 25 cm above the floor. Transfer latency (time to enter a closed arm with all four paws) was recorded with a 90 s cut-off. Initial latency was measured 45 min after scopolamine administration, and retention latency was assessed after 24 h and finally the inflexion ratio was calculated. Similarly, time spent in light and dark compartments by each animal was recorded by using a light/dark test apparatus. The apparatus comprised interconnected light and dark compartments. Mice were placed in the light chamber and observed for 5 minutes to record the time spent in each compartment. Testing was conducted for two consecutive days after treatment. Learning behavior was assessed using a hole board apparatus containing 16 equidistant holes. Animals were observed for 5 min, and the number of head dips was recorded. The detailed procedures along with protocols are explained in our previous studies [27,28,29].

### 2.9. Biochemical Studies

Once the behavioral studies were done on the 8th day of the treatment then all the animals were sacrificed by using chloroform and decapitated for brain isolation. Brains were weighed, rinsed with ice-cold saline, and homogenized (20 mg/mL) in ice-cold phosphate buffer (pH 7.4). The homogenate was centrifuged at 4 °C to remove debris, and the resulting supernatant was used for biochemical analyses. The homogenates were used to estimate the levels of two well-known enzymes, choline acetyl transferase (ChAT) and acetylcholinesterase (AChE). Similarly, the antioxidant enzymes catalase (CAT), reduced glutathione (GSH), malondialdehyde (MDA) and superoxide dismutase (SOD) were also quantified in the brain homogenates. The methods used for estimation of all these enzymes along with detailed procedures are available in our previously published studies [29].

### 2.10. Histopathological Studies

Histopathological studies were performed on mice brains. Two out of six mice from each group were specified for the histopathological studies. The mice were sacrificed by using chloroform once the treatment span was over. Brains were washed with normal saline and preserved in 10% buffered formalin. Individual slides were prepared by cutting the brain from the middle and by fixing it in paraffin. The frozen paraffin-embedded brain tissue was subjected to a microtome and slices of 5 μm thickness were made. A very fine slice from each group was first washed with ethanol and then rehydrated with distilled water and was put on a clean glass slide. The slides were stained by using hematoxylin–eosin dye and were properly fixed with xylene [30]. A digital microscope of resolution power (100 X) was used for microscopic study of slides. The photomicrograph of each slide was taken by a top-mounted microscopic camera and histopathological evaluation of all the slides was done by giving them a particular score for the presence of pathological indications like necrosis, inflammation, fibrosis, edema and neuronal degeneration as shown in Table 2. Finally, the histology activity index (HAI) of each photomicrograph was calculated by taking sum of all the scores outlined in Table 2 [31].

### 2.11. Statistical Analysis

Data were presented as mean ± standard error of the mean (SEM). Statistical analysis was performed using one-way ANOVA followed by Dunnett’s post hoc test in GraphPad Prism Version 8. Comparisons were conducted as follows: the amnesic control group was compared with the normal control group, and all treatment groups, except the vehicle control (empty HPSL), were compared with the amnesic control group. Finally, both experimental control groups were compared with the vehicle control group. A *p*-value < 0.05 was considered statistically significant.

## 3. Results

### 3.1. Characterization and Optimization of HPSL: A Physical Perspective

The particle size increases as the concentrations of heparin and pullulan increase. The largest particle size was observed for HPSL-3 (406.07 ± 19.33 nm), making it beneficial for drug delivery applications requiring stability and slower release rates. HPSL-4 (319.76 ± 21.57 nm) also showed a relatively large particle size, whereas HPSL-1 and HPSL-2 had smaller particle sizes of 189.73 ± 14.29 nm and 233.81 ± 39.64 nm, respectively. Regarding loading efficiency, HPSL-4 displayed the highest value (42.74 ± 6.02%), followed by HPSL-2 (39.16 ± 7.45%). HPSL-3 exhibited a moderate loading efficiency (30.91 ± 4.08%), indicating a balance between drug loading and other properties such as release profile and stability. HPSL-1 had the lowest loading efficiency (27.55 ± 6.89%).

The entrapment efficiency of HPSL-4 (93.67 ± 3.21%) was the highest among all formulations, suggesting effective drug encapsulation. Although HPSL-3 did not have the highest entrapment efficiency (73.53 ± 12.05%), it still demonstrated significant retention of the drug within the particles. HPSL-2 also showed a high entrapment efficiency (89.31 ± 7.74%), whereas HPSL-1 had the lowest value (67.22 ± 11.37%).

In terms of particle size distribution, HPSL-1 had the lowest polydispersity index (PDI) of 0.306 ± 0.025, indicating the most uniform size distribution. HPSL-3 exhibited a higher PDI (0.504 ± 0.061) compared to HPSL-1 and HPSL-2 (0.427 ± 0.014) but it was lower than that of HPSL-4 (0.596 ± 0.071). This suggests moderate uniformity, which may be acceptable depending on application requirements.

**Figure 1:**
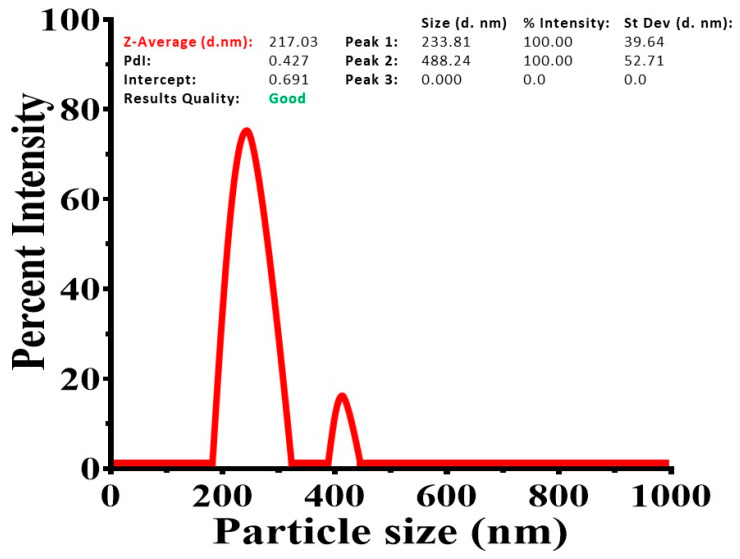
Determining the hydrodynamic diameter of silymarin–NS.

All formulations exhibited a negative zeta potential, which is significant for stability due to electrostatic repulsion. The zeta potential of HPSL-3 was the least negative (−23.72 ± 7.64 mV) among all formulations, which might enhance interactions with biological membranes. In comparison, HPSL-1, HPSL-2, and HPSL-4 had zeta potentials of −26.39 ± 4.14 mV, −29.88 ± 5.62 mV, and −25.69 ± 7.98 mV, respectively. The details are represented in Table 3 and Figure 1.

### 3.2. Scanning Electron Microscopy

**Figure 2:**
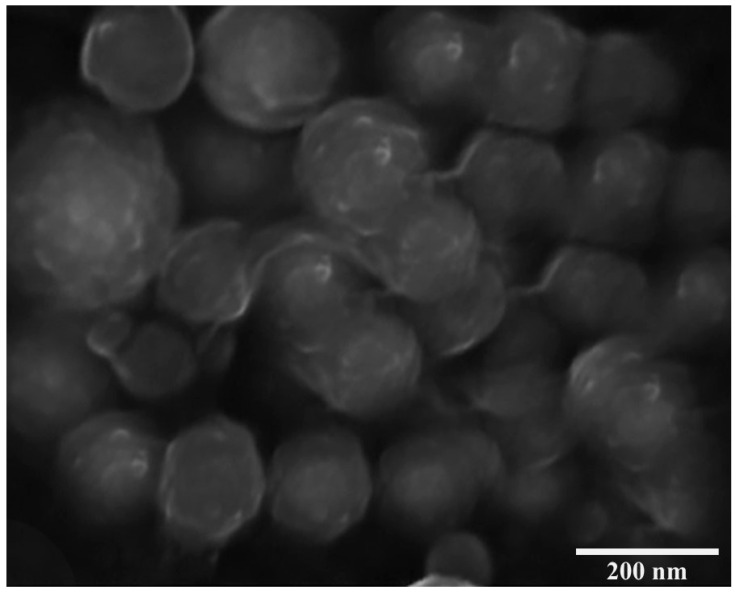
The physiochemical characterization of HPSL-2 was examined using SEM analysis.

The scanning electron microscopy (SEM) image presents a cluster of HPSL-2 nano-gels. The image is captured at a magnification power of 74,900×, revealing the complex details of the nano-gels (Figure 2).

### 3.3. Silymarin Release Kinetics

**Figure 3:**
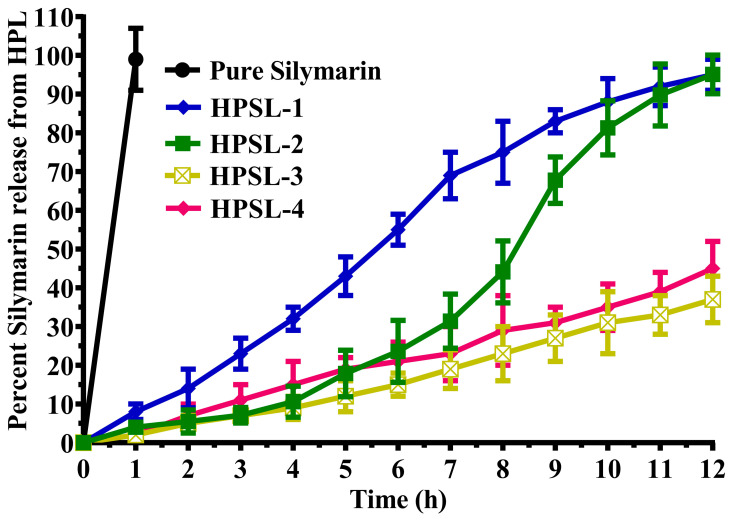
The in vitro release profile of HPSL nano-gels (HPSL-1, HPSL-2, HPSL-3, and HPSL-4) and pure silymarin evaluated over a 12-hour period. The data are expressed as mean ± SD (n = 3). The physical characterization results suggest that HPSL-2 is the most well-balanced and effective formulation for targeted drug delivery. With a mean particle size of 233.81 ± 39.64 nm, it hits a ‘sweet spot’ large enough to hold a sufficient amount of drug, yet small enough to allow better cellular uptake and easier penetration through biological barriers, unlike the bulkier HPSL-3 (406.07 nm). Although HPSL-4 shows slightly higher drug entrapment, HPSL-2 achieves an impressive 89.31 ± 7.74% entrapment efficiency and the highest loading efficiency among all formulations at 39.16 ± 7.45%. This high loading capacity ensures that a therapeutic dose can be delivered effectively while minimizing the amount of carrier, thereby optimizing the therapeutic potential of the silymarin nano-gel. HPSL-2 also demonstrates the strongest colloidal stability, with a zeta potential of −29.88 ± 5.62 mV, the highest absolute value among all formulations. This pronounced negative surface charge provides sufficient electrostatic repulsion to prevent particle aggregation, promoting longer shelf-life and stable circulation in vivo. Its PDI of 0.427 ± 0.014 indicates a more uniform size distribution compared to formulations with higher polymer concentrations, such as HPSL-4 (PDI 0.596), suggesting better reproducibility across batches. As a result, HPSL-2 is expected to provide an efficient and controlled release of silymarin, minimizing erratic burst release from smaller particles and incomplete release from overly dense matrices.

The in vitro release profile of silymarin from the HPSL-2 nano-gel is shown in Figure 3, where the cumulative percentage of silymarin released is plotted against time. The graph demonstrates a comparatively faster release rate of silymarin from the HPSL-2 nano-gel than from the pure drug. As expected, pure silymarin exhibited a faster dissolution in the release medium (water/PBS) due to the absence of any diffusion barrier. In contrast, drug release from the HPSL nano-gel was governed by matrix-controlled mechanisms, including diffusion through the polymeric network and polymer swelling/relaxation processes. Consequently, the nano-gel formulation demonstrated a comparatively slower and more controlled (sustained) release profile.

### 3.4. Findings of Behavioral Studies

The results of behavioral studies conducted by elevated plus maze indicated that the administration of animals with HPSL-2 nano-gel 40 mg/Kg produced significant (*p* ≤ 0.001) increases in the inflexion ratio as compared to scopolamine-treated hyperamnesic mice. Similarly, the findings of the hole-poking paradigm presented significantly (*p* ≤ 0.001) higher poking through holes by mice treated with HPSL-2 nano-gel 40 mg/Kg. The 3rd paradigm used was the light–dark test, the results of which indicated that the mice treated with HPSL-2 nano-gel retained their memory of exploration more efficiently and spent more time in the dark compartment as compared to other groups (piracetam-treated group, silymarin-treated group and scopolamine-treated mice), which lost their memory of finding the entrance hole and spent most of the time in the light area. Conversely, the animals treated with empty HPSL-2 (40 mg/kg) without silymarin showed poorer behavioral performance. They exhibited a lower inflexion ratio, made fewer hole-poking responses, and spent more time in the dark compartment compared to the experimental control groups. These findings suggest impaired learning, memory, and exploratory behavior in this group (Table 4, Table 5 and Table 6).

### 3.5. Findings of Biochemical Studies

The findings of biochemical studies conducted on mice brains indicated that the level of AChE was significantly reduced using HPSL-2 nano-gel while the ChAT level was found to be increased in mice treated with HPSL-2 nano-gel in comparison to scopolamine-treated animals. Similarly, antioxidant markers (CAT, GSH and SOD) increased significantly (*p* ≤ 0.001) after treatment with HPSL-2 nano-gel. The MDA level was lowered in mice treated with HPSL-2 nano-gel in comparison to the scopolamine control group. The mice treated with silymarin produced less efficient results in comparison to mice treated with HPSL-2 nano-gel. However, animals treated with the empty HPSL-2 nano-gel exhibited results closely comparable to those of the amnesic control group, as no significant improvement in biochemical markers was observed relative to the experimental control groups (Table 7).

### 3.6. Results of Histopathological Studies

**Figure 4:**
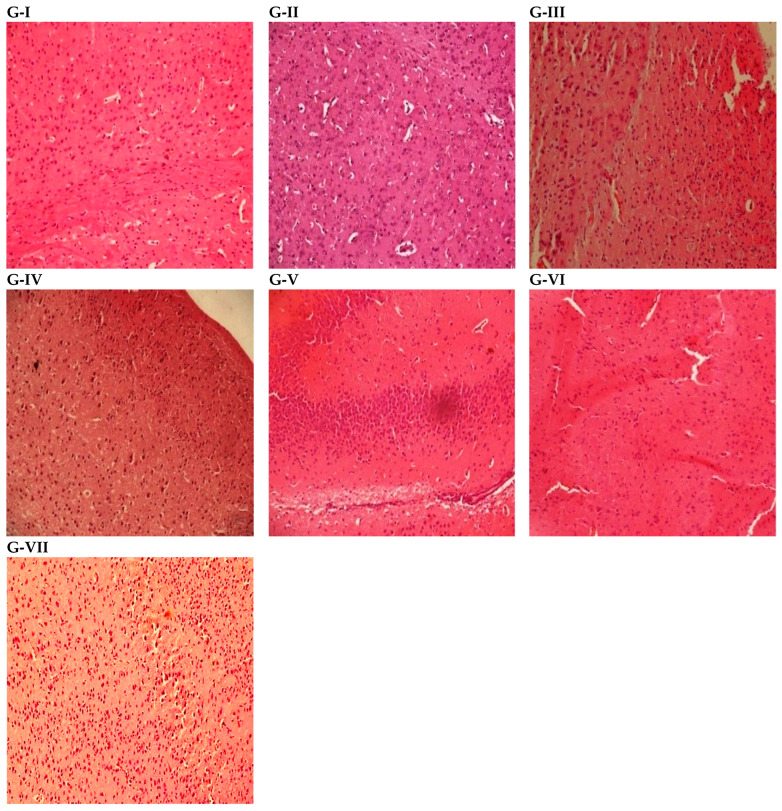
Photomicrographs of histopathological studies (hematoxylin and eosin staining, magnification power at 100×). (**G-I**) Normal Control, (**G-II**) Amnesic Control, (**G-III**) Standard Control, (**G-IV**) Silymarin Control, (**G-V**) Experimental Control Low Dose (HPSL-2 nano-gel 20 mg/Kg), (**G-VI**) Experimental Control High Dose (HPSL-2 nano-gel 40 mg/Kg) and (**G-VII**) Vehicle Control (Empty HPSL-2 nano-gel without Silymarin).

Histopathological studies of mice brains revealed significant findings following the treatment protocols. The animals exhibited pronounced inflammation and edema, with the highest histopathology activity index (HAI) score observed at 11 in the toxic control group. In contrast, the standard control group showed lower HAI scores compared to the toxic control group. Among the experimental groups, animals treated with silymarin showed HAI 7 in comparison to HPSL-2 nano-gel 20 mg/Kg and HPSL-2 nano-gel 40 mg/Kg groups which exhibited lower HAI scores of 5 and 4 respectively. However, the empty HPSL-2 nano-gel group exhibited a comparatively elevated HAI level (10) relative to the experimental control groups. The results are summarized in the accompanying Table 8 and Figure 4 for detailed reference.

## 4. Discussion

The prevalence of dementia is increasing worldwide and experts continuously predict significant future health burdens associated with this disorder. Individuals above sixty years of age are at higher risk [32]. There is a strong need to explore new remedies which may be helpful in the prevention of dementia. Considering the current research demand in this area and to overcome the challenges associated with drug delivery across the blood–brain barrier, we designed the present study to evaluate the benefits of an advanced, hybrid nano-carrier drug delivery system for the prevention and cure of dementia. Silymarin has been scientifically proven to have hepatoprotective activity and it is widely used in practice for the management of numerous hepatic dysfunctions [14]. Its efficacy against dementia is well supported by scientific evidence [16], however, poor aqueous solubility and limited ability to exert neuroprotective effects in the brain remain major challenges for researchers [17]. It exerts hepatoprotective effects by scavenging highly reactive free radicals along with detoxifying harmful toxins. It mitigates tissue injury by preventing the activation of certain proinflammatory mediators and cytokines. It stabilizes the hepatic cell membranes and prevents the leakage of enzymes into the blood. Moreover, its antifibrotic action and modulation of hepatic cell regeneration are two principle mechanisms by which it presents promising hepatoprotective action [33]. The protective effects of silymarin against scopolamine-induced dementia in rats are mediated through its potent antioxidant and anti-inflammatory properties, along with the enhancement of cholinergic neurotransmission in the brain [16].

In the present study, a heparin–pullulan liposomal nano-gel of silymarin was designed to enhance colloidal stability and promote effective penetration across the blood–brain barrier. The formulated HPSL-2 nano-gel of silymarin was subsequently evaluated for its modulatory effects on key brain enzymes, including choline acetyltransferase (ChAT), acetylcholinesterase (AChE), catalase (CAT), reduced glutathione (GSH), and superoxide dismutase (SOD).

The physical characterization of HPSL nano-gels revealed that increasing concentrations of heparin and pullulan led to larger particle sizes. Among the formulations, HPSL-2 showed an optimal particle size of 233.81 ± 39.64 nm, striking a balance between the smaller HPSL-1 and the larger HPSL-3 (406.07 nm). This size is large enough to act as a stable drug reservoir, yet small enough to facilitate efficient cellular uptake and penetration across biological barriers.

While HPSL-1 had the smallest particle size, HPSL-2 exhibited a well-controlled polydispersity index (0.427 ± 0.014), reflecting a uniform particle distribution ideal for reproducible drug delivery. All formulations showed negative zeta potentials, indicating good colloidal stability. Notably, HPSL-2 displayed the strongest surface charge (−29.88 ± 5.62 mV), the highest absolute value among the set, providing sufficient electrostatic repulsion to prevent particle aggregation and maintain long-term stability in biological media.

Based on these results, HPSL-2 was identified as the optimized formulation due to its well-balanced physicochemical properties. With a particle size of 233.81 ± 39.64 nm, it falls within an ideal range, being large enough to serve as a stable drug reservoir, yet considerably smaller than the bulkier HPSL-3 (406.07 nm), allowing for more efficient cellular uptake and improved penetration across biological barriers.

Importantly, HPSL-2 exhibited the highest loading efficiency (39.16 ± 7.45%) among all tested formulations, ensuring that a potent therapeutic dose can be delivered with minimal carrier material. This was complemented by a high entrapment efficiency (89.31 ± 7.74%), indicating that the majority of silymarin was successfully retained within the nano-gel matrix. Additionally, HPSL-2 demonstrated the strongest colloidal stability, with a zeta potential of −29.88 ± 5.62 mV, the highest absolute value in the set and providing sufficient electrostatic repulsion to prevent particle aggregation. Coupled with a controlled polydispersity index (0.427 ± 0.014), these characteristics support uniform batch-to-batch performance and a predictable, efficient drug release profile, making HPSL-2 the most promising candidate for targeted delivery applications.

In conclusion, the combination of adequate drug loading and entrapment efficiencies, optimal particle size, appropriate zeta potential, and acceptable uniformity makes HPSL-2 a favorable candidate for further experimentation in drug delivery systems.

The nano-gels exhibit a predominantly spherical morphology, which is a typical characteristic of successfully self-assembled polysaccharide-based nano-carriers [34]. This indicates that the self-assembly process of the HPSLs has led to the formation of well-defined, uniform structures.

The relatively smooth surface of the nano-gels suggests that the liposomal membrane is intact and free from significant irregularities. A key feature observed is the close packing of the nano-gels. This characteristic tight packing, combined with their internal network, is highly advantageous for achieving high drug loading capacity and efficient delivery [35].

The smooth surface, spherical morphology, and close packing recommend these nano-gels as appropriate platforms for efficient drug delivery [34,35]. Specifically, the HPSL carrier system is designed to enhance the neuroprotective efficacy of challenging drugs like silymarin, potentially leading to enhanced therapeutic outcomes, while direct confirmation of brain delivery requires further investigations [36]. Pullulan serves as a biocompatible backbone that facilitates nanoparticle formation [37] while heparin can contribute to targeted effects [36].

In conclusion, the SEM image confirms morphological features that propose a favorable platform for targeted drug delivery. However, as is common with novel nano-carrier systems, more research is required to fully analyze the improvement in potential therapeutic efficacy and safety of silymarin delivered via this HPSL nano-gel [35].

The enhanced release kinetics can be attributed to the nano-scale size and liposomal encapsulation of silymarin within the HPSL matrix, which significantly increase the effective surface area for diffusion and dissolution, a well-documented phenomenon in nano-carrier systems [38,39].

HPSL is a nano-gel comprising a cross-linked network of the natural polysaccharides heparin and pullulan. These polymers are widely recognized for their excellent biodegradability and biocompatibility, making them safe and effective for biomedical applications [40,41]. This inherent safety profile is a critical characteristic, suggesting a low risk of toxicity for potential clinical application.

The liposomal component of HPSLs is specifically designed to encapsulate silymarin, shielding it from premature degradation in the biological environment [42]. This protection ensures that a greater proportion of the drug remains intact until it reaches the target site. The synergistic combination of the protective liposomes and the tunable, biocompatible nano-gel network creates a drug delivery system that is both efficacious and safe. Therefore, the HPSL presents itself as a highly favorable candidate for the targeted delivery of silymarin and other challenging therapeutic compounds [38,42].

The R^2^ coefficient provides a quantitative measure for analyzing the kinetics and release patterns of silymarin from HPSL nano-gel [43]. The R^2^ value derived from first-order analysis (0.9438) indicates that the concentration of silymarin directly influences its release from the nano-gel within a specific timeframe. This suggests that HPSL nano-gel exhibits a concentration-dependent drug release profile. Additionally, the Peppas model yielded a high R^2^ value (0.9817, n = 0.865), indicating that the release mechanism of silymarin from HPSL nano-gel is governed by erosion, swelling, and non-Fickian super case II transport. These findings confirm that HPSL nano-gel facilitates sustained drug release, making it a promising system for controlled drug delivery applications [44]. The use of HPSL nano-gel in drug delivery offers several advantages. One of the key benefits is sustained drug release, as the nano-gel acts as a drug reservoir, gradually releasing the drug over an extended period. This controlled release mechanism ensures prolonged therapeutic effects while reducing the frequency of drug administration. Additionally, HPSL nano-gel enhances drug delivery by enabling targeted drug release at specific sites in the body. The presence of liposomes within the nano-gel protects the drug from premature degradation, ensuring its availability at the intended site for optimal therapeutic efficacy.

Another significant advantage of HPSL nano-gel is the reduction in drug-related side effects. By restricting the rapid absorption of the drug, the nano-gel minimizes the risk of toxicity, making the treatment safer for patients. Furthermore, HPSL nano-gel improves patient compliance by offering a more convenient mode of administration. Designed for both oral and intravenous delivery, HPSL nano-gel formulations provide flexibility in treatment, making drug therapy more accessible and user-friendly. In summary, HPSL nano-gel represents a novel drug delivery system with the potential to revolutionize therapeutic approaches by improving drug stability, targeting efficiency, and patient adherence to treatment.

The results of in vivo testing indicated that the treatment of mice with HPSL-2 nano-gel produced significant changes in their behavior. The inflexion ratio found by the elevated plus maze paradigm indicated a significant (*p* ≤ 0.001) increase in the inflexion ratio of mice treated with HPSL-2 nano-gel in comparison to negative and positive control groups. Similarly, the seeking behavior of mice was recorded to be improved in the HPSL-2 group as found by the hole board test paradigm. Our 3rd paradigm was the light–dark test which indicated that the animals of standard control and experimental control (treated with HPSL-2 nano-gel) spent the maximum time in the dark compartment as compared to amnesic control animals. These three paradigms are widely used to assess memory and retention capacity in experimental mice following treatment with a test substance [45]. Increased inflexion ratio indicated that animals learned the task effectively and also retained it. Similarly, increasing poking indicates retaining seeking behavior. The scopolamine-treated mice forgot how to find the passage to enter the dark compartment and they spent most of the time in the light area while HPSL-2 nano-gel and standard control drug groups retained the memory and they entered the dark area in less time. Thus, the behavioral studies indicated that animals treated with silymarin-loaded HPSL-2 nano-gel retained the learned tasks in contrast to the amnesic control animals. In all the behavioral paradigms, the mice given HPSL-2 nano-gel alone (G-VII) showed no noticeable changes compared with normal control animals (*p* > 0.05). Measures like memory retention in the elevated plus maze, head-poking in the hole board test, and time spent in light or dark compartments were all similar to the amnesic control. This shows that HPSL-2 nano-gel by itself does not affect learning, memory, or exploratory behavior. Therefore, the improvements seen in other treated groups are due to silymarin delivered by the nano-gel, not the nano-gel itself, which acts only as a safe, inactive carrier.

The biochemical studies performed on mice brain indicated that HPSL-2 significantly (*p* ≤ 0.001) increased antioxidant enzymes including CAT, SOD and GSH while the level of MDA (a marker for lipid peroxidation) in brain homogenates was significantly reduced in comparison to scopolamine-treated mice. This finding strongly suggested the potent antioxidant potential of HPSL-2 nano-gel for brain neurons. Pathophysiology of dementia suggests that the overloading of oxidizing radicals is one of the leading causes of neurodegeneration in the brain. Lipid peroxidation reactions are propagated by overburdening by hydroxyl free radicals and as a result the MDA level is increased as seen in scopolamine-intoxicated mice [46]. This also reflects the use of MDA as a valuable marker to assess the antioxidant activity of any substance. Similarly, our finding indicated a significant reduction in the level of natural antioxidants like CAT, SOD and GSH by the use of scopolamine. Thus, scopolamine promoted the oxidizing burden in mice brains which ultimately led to the apoptosis of neurons and loss of retention power. Previous studies have well proved that the activation of interleukin 1β and related cytokines by administration of scopolamine resulted in cholinergic dysfunctions in the hippocampus of the mouse brain and ultimately amnesia [47]. Our findings suggested significant (*p* ≤ 0.001) increases in the levels of CAT, SOD and GSH in brain homogenates of mice treated with silymarin as well as HPSL-2 nano-gel (Table 5). This increase in the level of antioxidants prevented the loss of cholinergic neurons and established the retention power as supported by the results of our behavioral studies (Table 2, Table 3 and Table 4). A high level of CAT is actually responsible for reductions of free peroxide species into molecular oxygen and prevents neuronal damage. Similarly, SOD neutralizes superoxide free radicals and establishes 1st line defense against oxidizing reactions. Increased levels of reduced GSH donate electrons to oxygen free radicals and hence protect the brain from damage [48,49].

Biochemical findings of two important brain enzymes, i.e., ChAT and AChE (Table 5), showed a significant difference between the amnesic control and test groups. The marked increase in the level of ChAT and the significant reduction in the level of AChE in test groups (GI-GVI) indicate the buildup of a high level of acetylcholine (ACh) in the mouse brain. Hence, the memory of treated mice may be enhanced by this phenomenon. The pathogenesis of dementia is well explained by the cholinergic hypothesis [50] which states that dysfunction of cholinergic neurotransmission in the hippocampus of the brain affects memory and cognition. Following this statement, our results support that the level of acetylcholine in mice brains is significantly increased in two ways and hence memory and cognition can be restored by the use of HPSL-2. It has also been proved by the previous studies showing that piracetam possesses strong antioxidant properties with improvement in mitochondrial functionality and stabilization of membrane fluidity in the hippocampus [51]. Thus, we used this as a reference memory enhancer drug in our study to compare the effectiveness of our tested substances. In biochemical testing, the HPSL-2 control group (G-VII) showed no significant changes (*p* > 0.05) compared with the amnesic control. Levels of AChE, ChAT, CAT, GSH, MDA, and SOD were all comparable, indicating that the nano-gel alone does not affect the cholinergic function or antioxidant defense in the mouse brain. Although ANOVA showed significant differences across all groups, post hoc tests confirmed that HPSL-2 alone did not alter any parameter. This confirms that the nano-gel is pharmacologically neutral and safe as a silymarin carrier.

The histopathological findings described in our study underscore the impact of scopolamine treatment on mice brains, revealing marked inflammation and edema, as indicated by an elevated histopathology activity index (HAI) score of 11 in the toxic control group. These observations align with previous research highlighting scopolamine’s neuroinflammatory effects. For instance, previous scientific studies have demonstrated similar outcomes, attributing scopolamine-induced neuroinflammation to its disruption of cholinergic neurotransmission and subsequent activation of inflammatory pathways in the brain [52].

Comparatively, the standard control group exhibited lower HAI scores, indicative of less severe histopathological changes, which is consistent with the expected outcomes in the absence of scopolamine-induced neurotoxicity. This supports the reliability of our experimental model and control measures in assessing the specific effects of scopolamine. Interestingly, within the experimental groups, a dosage-dependent response to HPSL-2 nano-gel treatment was observed in the scopolamine-induced neuroinflammation mice model, further demonstrating its beneficial effects on brain histopathology. Animals treated with a higher dose (40 mg/kg) of HPSL-2 nano-gel exhibited a significantly lower HAI score of 4, indicating a potentially protective or adaptive response, compared to a higher score of 7 in mice treated with plain silymarin. This suggests that the HPSL-2 nano-gel enhanced the neuroprotective efficacy of silymarin, effectively preventing neural damage, whereas unformulated silymarin showed limited therapeutic effects. Moreover, the observed dose-dependent therapeutic effect is noteworthy and warrants further investigation to elucidate the underlying mechanisms. The histopathology results also clearly described that the brains of mice treated with HPSL-2 nano-gel alone showed no improvement compared with the amnesic control group. Tissue texture and HAI scores were all similar, confirming that the nano-gel does not have any neuroprotective effects by itself. This supports its role as a safe and biologically inert carrier, with the protective effects primarily attributed to the delivered silymarin rather than the nano-gel system.

Overall, our findings contribute to the growing body of literature on scopolamine-induced neuroinflammation and highlight the complex interplay between dose-dependent effects, neuroinflammatory responses, and the histopathological outcomes associated with HPSL-2 nano-gel treatment. These results suggest the potential therapeutic relevance of silymarin-loaded HPSL-2 nano-gel in managing neuroinflammatory conditions linked to cholinergic dysfunction. The physicochemical characterization demonstrated that the optimized HPSL-2 nano-gel exhibited appropriate particle size, acceptable polydispersity index, and satisfactory stability under normal storage conditions. However, a limitation of the present study is the absence of extended stability evaluation under physiological conditions, particularly in biologically relevant media such as phosphate-buffered saline (PBS). Serum components may influence surface properties, particle size, and aggregation behavior of the nano-gel through protein corona formation. Therefore, future studies should evaluate particle size and polydispersity index (PDI) changes over 24–48 h in serum-containing media to better understand in vivo stability. Further investigations will also focus on systematic stability assessment under simulated physiological conditions to enhance the translational potential of this formulation.

## 5. Conclusions

The heparin–pullulan liposomal nano-gel (HPSL-2) with silymarin improved memory, behavioral performance, and biochemical markers in a scopolamine-induced dementia model, demonstrating potential neuroprotective effects. The optimized HPSL-2 formulation provided sustained drug release, preserved cholinergic function, and restored antioxidant enzyme levels, outperforming unformulated silymarin. Importantly, the HPSL-2 control group showed no significant effects on behavioral, biochemical or histopathological parameters, confirming that the nano-gel itself is pharmacologically inert and acts solely as a carrier for silymarin. While these findings are encouraging, the study is limited by the lack of pharmacokinetic data, simplified in vitro models, limited empty carrier controls and the absence of extended stability evaluation under physiological conditions, particularly in biologically relevant media such as PBS. Future studies should investigate pharmacokinetics, mechanisms of action, and efficacy in advanced preclinical models to support potential clinical applications.

## Figures and Tables

**Table 1:** Study design along with treatment protocols for assessment of behavioral and biochemical parameters.

Group Number	Group Details	Treatment	
		Day 1–6	Day 7th
G-I	Normal Control	10 mL/Kg/p.o. normal saline	10 mL/Kg/p.o. normal saline
G-II	Amnesic Control	10 mL/Kg/p.o. 5% CMC	10 mg/Kg/p.o. scopolamine
G-III	Standard Control	200 mg/Kg/p.o. piracetam	200 mg/Kg/p.o. piracetam
G-IV	Silymarin Control	200 mg/Kg/p.o. silymarin	10 mg/Kg/p.o. scopolamine + 200 mg/Kg/p.o. silymarin
G-V	Experiment Control (low dose)	20 mg/Kg/p.o. HPSL-2 nano-gel	10 mg/Kg/p.o. scopolamine + 20 mg/Kg/p.o. HPSL-2 nano-gel
G-VI	Experiment Control (high dose)	40 mg/Kg/p.o. HPSL-2 nano-gel	10 mg/Kg/p.o. scopolamine + 40 mg/Kg/p.o. HPSL-2 nano-gel
G-VII	Vehicle Control	40 mg/Kg/p.o. empty HPSL-2 nano-gel without silymarin	10 mg/Kg/p.o. scopolamine + 40 mg/Kg/p.o. empty HPSL-2 nano-gel without silymarin

Note: HPSL-2 = heparin–pullulan silymarin liposome. The doses of scopolamine and piracetam were prepared by dissolving them in normal saline while silymarin was suspended in 5% carboxymethyl cellulose (CMC). The behavioral studies were performed two hours after dosing the animals on day 7 and also on day 8. After that the animals were sacrificed and their brains were preserved for biochemical studies.

**Table 2:** Calculation of histology activity index (HAI) of photomicrographs of mice brains after histopathology studies.

Indication	Scoring	Grading
Necrosis	0	Absence
	1	Minor
	2	Modest
	3	Marked
Inflammation	0	Absence
	1	Minor
	2	Modest
	3	Marked
Fibrosis	0	Absence
	1	Minor
	2	Modest
	3	Marked
Edema	0	Absence
	1	Minor
	2	Modest
	3	Marked
Degeneration	0	Absence
	1	Minor
	2	Modest
	3	Marked

Note: Greater HAI values represent more damage.

**Table 3:** Composition and physicochemical characterization of Silymarin-loaded Heparin–Pullulan Self-assembled Liposomes (HPSLs). Values for particle size, PDI, and zeta potential are expressed as Mean ± SD (n = 3).

	HPSL-1	HPSL-2	HPSL-3	HPSL-4
Silymarin (% *w*/*v*)	0.5	0.5	0.5	0.5
Heparin (% *w*/*v*)	1	1	2	2
Pullulan (% *w*/*v*)	1	2	1	2
Particle Size Mean ± SD (nm)	189.73 ± 14.29	233.81 ± 39.64	406.07 ± 19.33	319.76 ± 21.57
Loading Efficiency	27.55 ± 6.89	39.16 ± 7.45	30.91 ± 4.08	42.74 ± 6.02
Entrapment Efficiency	67.22 ± 11.37	89.31 ± 7.74	73.53 ± 12.05	93.67 ± 3.21
PDI Mean ± SD	0.306 ± 0.025	0.427 ± 0.014	0.504 ± 0.061	0.596 ± 0.071
Zeta Potential Mean ± SD (mV)	−26.39 ± 4.14	−29.88 ± 5.62	−23.72 ± 7.64	−25.69 ± 7.98

**Table 4:** Effect of silymarin on transfer latencies and inflexion ratio in albino mice.

Groups	Initial Transfer Latency L_1_ (s)	Retention Transfer Latency L_2_ (s)	Inflexion Ratio I.R = L_1_ − L_2_/L_2_
G-I	21.00 ± 0.73	17.83 ± 1.13	0.18 ± 0.04
G-II	70.50 ± 2.86 ^π^	85.16 ± 1.95 ^π^	−0.16 ± 0.04 ^µ^
G-III	44.83 ± 1.40 ^α^	37.66 ± 1.25 ^α^	0.20 ± 0.05 ^γ^
G-IV	19.66 ± 0.88 ^α^	16.50 ± 0.76 ^α^	0.19 ± 0.03 ^γ^
G-V	35.50 ± 1.60 ^α€^	25.66 ± 1.35 ^α€^	0.40 ± 0.11 ^α¥^
G-VI	41.16 ± 1.24 ^α€^	24.33 ± 1.47 ^α€^	0.71 ± 0.10 ^α€^
G-VII	71.33 ± 2.59	84.17 ± 2.44	−0.14 ± 0.04

Note: G-II was compared to G-I and superscripts ^π^ and ^µ^ represent level of significance where ^π^ = *p* ≤ 0.001 and ^µ^ = *p* ≤ 0.05. All other groups (G-III to G-VI) were compared to G-II and superscripts ^α^ and ^γ^ represent level of significance, where ^α^ = *p* ≤ 0.001 and ^γ^ = *p* ≤ 0.05. G-V and G-VI were compared to G-VII and superscripts ^€^, and ^¥^ represent level of significance, where ^€^ = *p* ≤ 0.001 and ^¥^ = *p* ≤ 0.05. Significant differences were observed among groups for initial transfer latency L_1_, (F(6, 35) = 138.5, *p* < 0.001), retention transfer latency L_2_ (F(6, 35) = 369, *p* < 0.001) and inflexion ratio (F(6, 35) = 19.19, *p* < 0.001).

**Table 5:** Effect of silymarin on number of hole pokings by albino mice in hole board paradigm.

Groups	Day 1	Day 2
	No of Pokings in 5 min	No of Pokings in 5 min
G-I	45.83 ± 1.35	40.33 ± 1.28
G-II	22.33 ± 1.58 ^π^	24.83 ± 1.55 ^π^
G-III	40.66 ± 1.35 ^α^	39.16 ± 1.66 ^α^
G-IV	38.66 ± 1.83 ^α^	34.66 ± 2.29 ^β^
G-V	41.00 ± 1.93 ^α€^	39.16 ± 2.27 ^α🗶£^
G-VI	50.16 ± 2.34 ^α€^	43.66 ± 1.76 ^α🗶€^
G-VII	23.34 ± 1.11	24.71 ± 1.52

Note: G-II was compared to G-I and superscript ^π^ represents level of significance as ^π^ = *p* ≤ 0.001. All other groups (G-III to G-VI) were compared to G-II and superscripts ^α^ and ^β^ represent level of significance where ^α^ = *p* ≤ 0.001 and ^β^ = *p* ≤ 0.01. G-V and G-VI were compared to G-VII and superscripts ^€^, ^£^ and ^🗶^ represent level of significance where ^€^ = *p* ≤ 0.001, ^£^ = *p* ≤ 0.01 and ^🗶^ = *p* > 0.05. One-way ANOVA revealed a significant effect of treatment on head-poking behavior on Day 1 (F(6, 35) = 39.85, *p* < 0.0001) and Day 2 (F(6, 35) = 17.93, *p* < 0.0001).

**Table 6:** Effect of silymarin on time spent by albino mice in light and dark compartment models.

Groups	Day 1		Day 2	
	Time Spent in Dark Compartment (s)	Time Spent in Light Compartment (s)	Time Spent in Dark Compartment (s)	Time Spent in Light Compartment (s)
G-I	247.66 ± 1.97	52.33 ± 1.97	248.50 ± 4.10	51.50 ± 4.10
G-II	112.33 ± 5.82 ^π^	187.66 ± 5.82 ^π^	089.83 ± 4.52 ^π^	210.16 ± 4.51 ^π^
G-III	233.66 ± 2.57 ^α^	66.33 ± 2.57 ^α^	239.66 ± 4.82 ^α^	57.00 ± 3.67 ^α^
G-IV	201.66 ± 3.06 ^α^	98.33 ± 3.06 ^α^	241.16 ± 3.60 ^α^	58.83 ± 3.60 ^α^
G-V	240.50 ± 3.08 ^α€^	59.50 ± 3.08 ^α€^	247.66 ± 2.57 ^α€^	52.33 ± 2.57 ^α€^
G-VI	254.66 ± 5.42 ^α€^	45.33 ± 5.42 ^α€^	258.66 ± 3.98 ^α€^	41.33 ± 3.98 ^α€^
G-VII	116.17 ± 4.99	183.83 ± 4.99	090.17 ± 4.55	209.83 ± 4.55

Note: G-II was compared to G-I and superscripts ^π^ represents level of significance as *p* ≤ 0.001. All other groups (G-III to G-VI) were compared to G-II and superscript ^α^, represents level of significance as *p* ≤ 0.001. G-V and G-VI were compared to G-VII and superscript ^€^ represents level of significance as = *p* ≤ 0.001. One-way ANOVA showed significant differences among groups for time spent in the dark compartment on Day 1 (F(6, 35) = 225.3, *p* < 0.0001) and Day 2 (F(6, 35) = 354.6, *p* < 0.0001) and for time spent in the light compartment on Day 1 (F(6, 35) = 225.3, *p* < 0.0001) and Day 2 (F(6, 35) = 389.8, *p* < 0.0001).

**Table 7:** Biochemical estimation of cholinergic enzymes along with antioxidant markers in mice brains.

Groups	AChE (µmol/min/mg)	ChAT (µmol/min/mg)	CAT (U/mg of Protein)	GSH (nmol/mg of Protein)	MDA (nmol/h/g)	SOD (U/mg of Protein)
G-I	04.52 ± 0.26	12.29 ± 1.01	01.48 ± 0.16	40.75 ± 0.89	01.62 ± 0.12	25.88 ± 0.93
G-II	09.28 ± 0.37 ^π^	08.08 ± 0.82 ^ns^	00.62 ± 0.03 ^π^	18.12 ± 0.34 ^π^	07.05 ± 0.35 ^π^	08.60 ± 0.26 ^π^
G-III	05.02 ± 0.27 ^α^	15.85 ± 1.21 ^α^	01.34 ± 0.07 ^α^	41.34 ± 1.09 ^α^	02.80 ± 0.20 ^α^	23.52 ± 0.81 ^α^
G-IV	06.90 ± 0.33 ^α^	12.64 ± 0.73 ^γ^	01.45 ± 0.14 ^α^	42.02 ± 1.06 ^α^	03.16 ± 0.19 ^α^	22.69 ± 0.72 ^α^
G-V	05.60 ± 0.33 ^α€^	13.68 ± 1.08 ^β🗶^	01.81 ± 0.06 ^α€^	47.92 ± 0.78 ^α€^	02.88 ± 0.22 ^α€^	24.67 ± 0.79 ^α€^
G-VI	04.09 ± 0.26 ^α€^	17.93 ± 1.14 ^α🗶£^	01.84 ± 0.07 ^α€^	48.22 ± 0.96 ^α€^	02.71 ± 0.15 ^α€^	25.06 ± 0.97 ^α€^
G-VII	09.69 ± 0.30	08.30 ± 0.80	00.63 ± 0.04	18.58 ± 0.26	07.62 ± 0.38	08.38 ± 0.37

Note: G-II was compared to G-I and superscripts ^π^ and ^ns^ represent level of significance where ^π^ = *p* ≤ 0.001 and ^ns^ = *p* > 0.05. All other groups (G-III to G-VI) were compared to G-II and superscripts ^α^, ^β^ and ^γ^ represent level of significance where ^α^ = *p* ≤ 0.001, ^β^ = *p* ≤ 0.01 and ^γ^ = *p* ≤ 0.05. G-V and G-VI were compared to G-VII and superscripts ^€^, ^£^ and ^🗶^ represent level of significance where ^€^ = *p* ≤ 0.001, ^£^ = *p* ≤ 0.01 and ^🗶^ = *p* > 0.05. One-way ANOVA demonstrated a significant effect of treatment on acetylcholinesterase (AChE) activity (F(6, 35) = 53.54, *p* < 0.0001), choline acetyltransferase (ChAT) activity (F(6, 35) = 13.45, *p* < 0.0001), catalase (CAT) activity (F(6, 35) = 26.44, *p* < 0.0001), reduced glutathione (GSH) levels (F(6, 35) = 239.8, *p* < 0.0001), malondialdehyde (MDA) levels (F(6, 35) = 88.21, *p* < 0.0001), and superoxide dismutase (SOD) activity (F(6, 35) = 115.4, *p* < 0.0001).

**Table 8:** Histology activity index (HAI) calculated from histopathological slides.

Groups	Relevant Score of Histopathological Lesions					
	Necrosis	Inflammation	Fibrosis	Edema	Degeneration	HAI
G-I	0	0	0	0	0	0
G-II	1	3	2	3	2	11
G-III	0	1	1	2	0	4
G-IV	1	2	1	2	1	7
G-V	0	2	0	2	1	5
G-VI	1	1	0	1	1	4
G-VII	2	1	2	3	2	10

## Data Availability

All data generated and analyzed in this study are included in this article. Additional data are available from the corresponding author upon reasonable request.
